# The landscape of transposable elements in the finished genome of the fungal wheat pathogen *Mycosphaerella graminicola*

**DOI:** 10.1186/1471-2164-15-1132

**Published:** 2014-12-17

**Authors:** Braham Dhillon, Navdeep Gill, Richard C Hamelin, Stephen B Goodwin

**Affiliations:** Department of Forest and Conservation Sciences, 2424 Main Mall, Vancouver, BC V6T 1Z4 Canada; Department of Botany, Beaty Biodiversity Centre, 2212 Main Mall, Vancouver, BC V6T 1Z4 Canada; Natural Resources Canada, Laurentian Forestry Centre, 1055 du PEPS, Stn. Sainte-Foy, P.O. Box 10380, Quebec, QC G1V 4C7 Canada; USDA-ARS, Crop Production and Pest Control Research Unit, Purdue University, 915 W. State Street, West Lafayette, Indiana 47907-2054 USA

## Abstract

**Background:**

In addition to gene identification and annotation, repetitive sequence analysis has become an integral part of genome sequencing projects. Identification of repeats is important not only because it improves gene prediction, but also because of the role that repetitive sequences play in determining the structure and evolution of genes and genomes. Several methods using different repeat-finding strategies are available for whole-genome repeat sequence analysis. Four independent approaches were used to identify and characterize the repetitive fraction of the *Mycosphaerella graminicola* (synonym *Zymoseptoria tritici*) genome. This ascomycete fungus is a wheat pathogen and its finished genome comprises 21 chromosomes, eight of which can be lost with no obvious effects on fitness so are dispensable.

**Results:**

Using a combination of four repeat-finding methods, at least 17% of the *M. graminicola* genome was estimated to be repetitive. Class I transposable elements, that amplify via an RNA intermediate, account for about 70% of the total repetitive content in the *M. graminicola* genome. The dispensable chromosomes had a higher percentage of repetitive elements as compared to the core chromosomes. Distribution of repeats across the chromosomes also varied, with at least six chromosomes showing a non-random distribution of repetitive elements. Repeat families showed transition mutations and a CpA → TpA dinucleotide bias, indicating the presence of a repeat-induced point mutation (RIP)-like mechanism in *M. graminicola*. One gene family and two repeat families specific to subtelomeres also were identified in the *M. graminicola* genome. A total of 78 putative clusters of nested elements was found in the *M. graminicola* genome. Several genes with putative roles in pathogenicity were found associated with these nested repeat clusters. This analysis of the transposable element content in the finished *M. graminicola* genome resulted in a thorough and highly curated database of repetitive sequences.

**Conclusions:**

This comprehensive analysis will serve as a scaffold to address additional biological questions regarding the origin and fate of transposable elements in fungi. Future analyses of the distribution of repetitive sequences in *M. graminicola* also will be able to provide insights into the association of repeats with genes and their potential role in gene and genome evolution.

**Electronic supplementary material:**

The online version of this article (doi:10.1186/1471-2164-15-1132) contains supplementary material, which is available to authorized users.

## Background

*Mycosphaerella graminicola* (synonym *Zymoseptoria tritici*, the causal agent of septoria tritici blotch, STB) poses a worldwide threat to wheat production, with yield losses of up to 30-40% or more during years with severe epidemics [1]. Although the use of fungicides and deployment of resistant wheat cultivars can help to contain *M. graminicola* losses in the field, breeding for resistance to STB has been slow and the resistance often is not durable [2]. With the rapid evolution of fungicide resistance in *M. graminicola* populations [3, 4] and failure of resistance genes in the field [2], there is an urgent need for improved measures to control STB.

Toward this end, availability of the *M. graminicola* genome, sequenced to completion by the Department of Energy - Joint Genome Institute (DOE-JGI) [5], is a valuable resource that may be utilized for developing better disease-control strategies. This can be achieved by identifying and characterizing the genomic components that may have an effect on the disease-causing abilities of the pathogen. Besides specific genes involved in pathogenicity and host specificity, intergenic regions and repetitive sequences, especially transposable elements (TEs), also influence the structure, function and regulation of genes.

Repetitive sequences are those that exist more than once in a genome, and are now known to be common features of eukaryotic genomes. Repetitive sequences include gene families, pseudogenes, segmental duplications, tandem repeats and transposable elements. Transposable elements, also known as mobile elements, are a special class of repetitive sequences that can move from one locus to another in a genome, either encoded proteins required for their own movement (autonomous TEs) or dependent on other autonomous elements for their movement (non-autonomous TEs). During the process of TE integration at a new genomic site, a few nucleotides flanking the new insertion site are duplicated creating a target site duplication (TSD), which is a signature for TE insertion/excision [6].

TEs can be divided into two main categories based on their mode of replication: Class I TEs or Retrotransposons; and Class II TEs or DNA transposons that also include the Miniature Inverted-repeat Transposable Elements (MITEs). Retrotransposons typically include coding sequences for several proteins including a reverse transcriptase that transcribes the RNA to a cDNA, which is integrated back into the genome, thereby following a copy-paste mechanism to move to a new genomic location. Retrotransposons can be further classified as Long Terminal Repeat (LTR) retrotransposons, which carry long terminal repeats at both ends, and Non-LTR retrotransposons, that lack LTRs but have a poly-A tail at their 3’ end. Class II (DNA-based) transposons, on the other hand, follow a cut-paste mechanism and move to a new genomic location without an RNA intermediate. DNA transposons typically are delimited by terminal inverted repeats (TIRs) and encode a transposase domain. Transposon-encoded transposase recognizes the TIRs, excises the element and integrates it into a new location [6]. Helitrons and cryptons are also classified as DNA transposons although they lack the traditional TIRs. Occurrence of distinct structural features and protein domains can be used to identify and distinguish between the different classes of TEs in a genome [6].

Since their discovery during the late 1940s by Barbara McClintock [7], the perceived importance of repetitive sequences has undergone a fundamental change from being considered inert components of the genome to drivers of genome evolution [8]. Identification of TEs is important to understand their functional significance, as they have the ability to alter the function and structure of the genome. For example, comparative genome analysis revealed that the three-fold genome size expansion of the oomycete *Phytophthora infestans* as compared to *P. ramorum* was mediated by TEs [9], altering the genome structure by creating gene-rich islands separated by vast expanses of repetitive sequences. Besides affecting the genome structure, TEs have the ability to create new genes [8, 10] and to modulate the function of existing genes to create new phenotypes [11]. An example of the latter phenomenon has already been documented in the *M. graminicola* genome, where a single-copy DNA methyltransferase gene was duplicated into a subtelomeric region and then amplified among the telomeres to a dozen copies, all of which were subsequently recognized by the repeat-induced point mutation (RIP) machinery and inactivated, including the original copy [12]. This led to a loss of cytosine methylation in *M. graminicola*, although the RIP machinery appears to be intact [12].

A survey of the completely sequenced *M. graminicola* genome was done to identify and categorize repetitive sequences, especially TEs, and to determine their chromosomal locations, both as independent and nested insertions. These data, when combined with the genome annotation [5], will help determine the association of TEs with the genic regions and further our understanding of TE-mediated processes that may be involved in the regulation of gene function.

## Results

### Identification of repetitive sequences

The 39.7-Mb genome of *M. graminicola* was sequenced to completion by the DOE-JGI; telomere-to-telomere sequence information is available for all but chromosome 18, which is missing two gaps of unclonable DNA (sizes of 1.4 and 4.5 kb), and chromosome 21, which is missing one telomere [13]. With only three gaps, it is the most finished genome for a filamentous fungus and comprises 21 chromosomes that carry 10,952 predicted genes [13]. The chromosomes have been arranged and named in order of their decreasing lengths. The *M. graminicola* genome is highly plastic as it can randomly lose up to eight of the smallest chromosomes (numbers 14–21), aptly labeled as dispensable [14].

**Figure 1:**
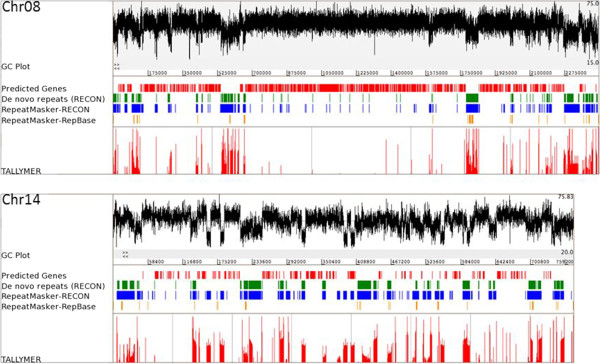
**Repetitive content on representative core (Chromosome 8) and dispensable (Chr. 14) chromosomes of** ***Mycosphaerella graminicola*** **.** Repetitive sequences in the *M. graminicola* genome were identified using four repeat-finding methods: RepeatMasker (RM), RECON, RepeatScout and TALLYMER. In addition to the default RepBase Update repeat library, two custom repeat libraries were used with RM. These custom repeat libraries contained repetitive elements identified by RECON and RepeatScout. Percent GC content was plotted for each chromosome. A sharp decrease in percent GC content coincides with the occurrence of repetitive sequences on both chromosomes. Different methods can be compared for repeat coverage on both chromosomes. RM-RepBase Update library (yellow bars) had the poorest coverage of repeats. The RM-RECON library (blue bars) performed better than RECON (green bars) alone. RepeatScout output is not shown here. The performance of TALLYMER (bottom red bars) was equivalent or better than that of the RM-RECON combination. Predicted genes are indicated by red bars.

**Figure 2:**
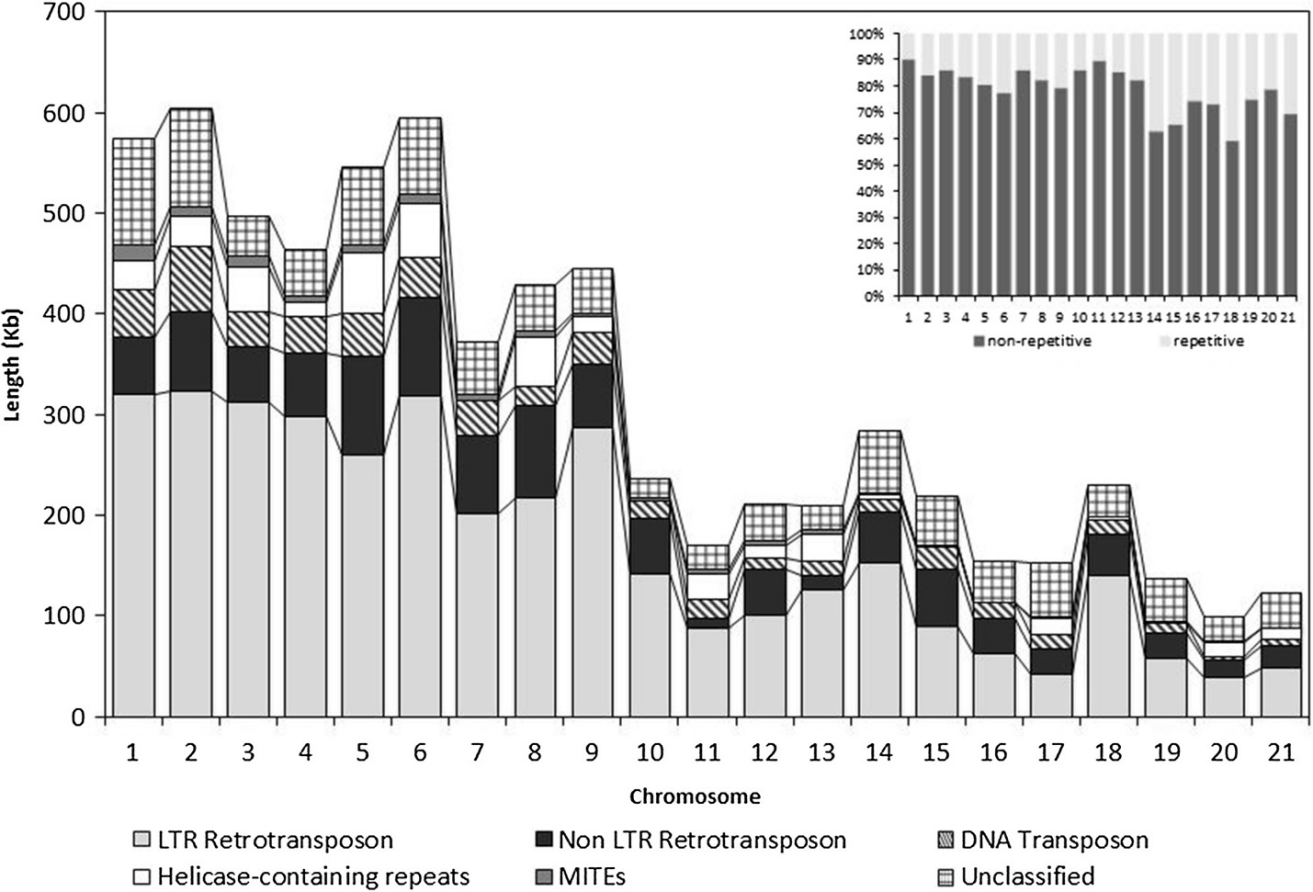
**The distribution of different classes of repetitive sequences across the** ***Mycosphaerella graminicola*** **chromosomes.** The contributions of six major repeat classes to the total repetitive content on each *M. graminicola* chromosome are shown. Inset: The percent repetitive content on each *M. graminicola* chromosome. The dispensable chromosomes have a higher percentage of repetitive sequences as compared to the core chromosomes.

Four strategies were employed to estimate the repetitive content of the *M. graminicola* genome. Initially, only 1.1% of the *M. graminicola* genome was identified as being repetitive when using RepeatMasker (RM) [15] with the default RepBase Update [16] repeat library containing fungal-specific repeats. This led us to identify repeats *ab initio* to compile and annotate a custom repeat library, for which RECON [17] was used. When this custom repeat library was used with RM [15], discovery of divergent elements increased the estimated repetitive content of the *M. graminicola* genome to 16.7% (6.7 Mb) (Table 1). This repetitive fraction does not reflect the tandem repeats (low-complexity and simple sequence) and low-copy repetitive families with fewer than ten repeats per family; there were 2,500 low-copy repeat families in total that accounted for 2,279,215 bp (5.7%) of the *M. graminicola* genome. RepeatScout [18], another tool for *de novo* repeat identification, was used to create a second custom repeat library which, in conjunction with RM [15], identified 18.7% (7.4 Mb) of the *M. graminicola* genome as repetitive (Table 1). A k-mer based approach, TALLYMER [4], was also used to identify and plot repeats across all of the chromosomes. The repeats predicted by different methods have been shown as separate tracks (Figure 1) for an essential (chromosome 8) and a dispensable chromosome (chromosome 14). The core chromosomes contain 88% of the genome and 79% of the repetitive fraction, whereas, the remaining 21% of the repeats are present on the 12% of the genome contained in the dispensable chromosomes. In general, the dispensable set of chromosomes had a statistically significantly higher (t = 6.1292, P < 0.0001) repetitive content compared to the core-chromosome set (Figure 2).

**Table 1:** **Predicted repetitive content in the** ***Mycosphaerella graminicola*** **genome**

Method	Repetitive bases	Percent repetitive
RECON, parsed	4,629,313	11.7
RepeatMasker - RepBase Update	467,778	1.2
RepeatMasker - RECON, parsed	6,921,597	17.4
RepeatMasker - RECON, parsed, nolow*	6,634,996	16.7
RepeatMasker - RepeatScout	7,400,585	18.6

*RepeatMasker nolow option, does not mask the low-complexity DNA or simple repeats.

### Repeat annotation

**Figure 3:**
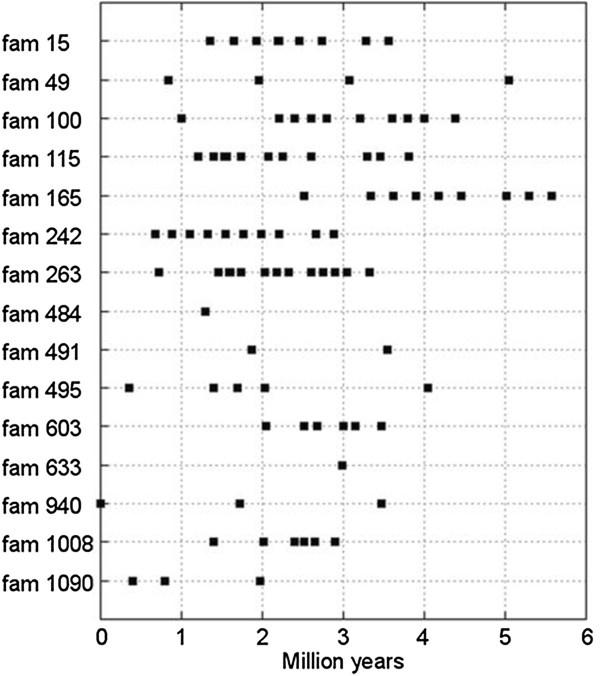
**LTR retrotransposon insertion age in the** ***Mycosphaerella graminicola*** **genome.** Insertion age for 149 elements from different *M. graminicola* LTR retrotransposon families was calculated using the nucleotide divergence between intact LTR pairs. The probable insertion age of each element is represented by square dots.

The repeat families identified by RECON [17] were categorized into six major classes (Table 2). Based on the repeat copy number, 105 families of high-copy repeats (10 or more copies in the genome) were annotated in detail in the *M. graminicola* genome. However, based on their distribution in the genome and sequence overlap, 12 families were merged with other families, decreasing the effective number of families to 93 with copy numbers ranging from 7 to 272 (Additional file 1: Table S1). Among the 2,500 low-copy repetitive families, only 125 families occupying 525,399 bp (1.3%) of the *M. graminicola* genome could be annotated (Additional file 2: Table S2). Among the different repeat classes, retrotransposons were the most common in the *M. graminicola* genome. In total, 21 LTR retrotransposon and four non-LTR retrotransposon families were identified. Class I TEs (both LTR and non-LTR retrotransposons) in total comprised 70.5% of the repetitive fraction in the genome. LTRs and TSDs could only be determined for 15 LTR retrotransposon families; the remaining 6 families had other characteristics of LTR retrotransposons except for the LTR. LTR lengths ranged from 110 to 378 bp and length of the TSDs varied from 4–5 bp. Average insertion age for the LTR retrotransposons was estimated to be 2.4 ± 1 Million years (My), with the oldest insertion event clocked at 5.6 My (Figure 3).

**Table 2:** **Classification of** ***Mycosphaerella graminicola*** **repetitive families identified** ***de novo*** **by using RECON**

Class	Repeat class (# families)	Repeat type	Total length (bp)*	Percent of repetitive content	Percent of genome
Class I	LTR Retrotransposon (21)	Ty1-Copia	1,039,062	15.7	2.6
		Ty3-Gypsy	1,803,457	27.2	4.5
		Unclassified	766,193	11.5	1.9
***Subtotal***			***3,608,712***	***54.4***	***9.1***
	Non LTR Retrotransposon (4)	Tad1-like	697,083	10.5	1.8
		Unclassified	371,533	5.6	0.9
***Subtotal***			***1,068,616***	***16.1***	***2.7***
Class II	DNA Transposon (15)	CACTA En/Spm	78,909	1.2	0.2
		hAT	70,346	1.1	0.2
		Tc1-Mariner	58,977	0.9	0.1
		Tc5/Pogo	44,941	0.7	0.1
		Unclassified	228,727	3.4	0.6
	MITEs (13)		98,726	1.5	0.2
	Helitron (3)		390,961	5.9	1.0
***Subtotal***			***971,587***	***14.6***	***2.4***
Unclassified	Unclassified (37)	Unclassified	986,081	14.9	2.5
**Total**			**6,634,996**		**16.7**

*These repeat estimates were calculated using families containing at least 10 members.

Unclassified repeats, those with structural features characteristic of the major groups but with no similarity to protein domains or structural features associated with sub-groupings of known repeats, was the next major category. Thirty-seven families of unclassified repeats were identified that occupied 14.9% of the repetitive fraction.

Fifteen families of non-MITE DNA transposons, thirteen MITE families and three families of helitrons (helicase domain-containing repeats) were also identified in the genome. Helitrons are atypical DNA transposons as they lack TIRs and do not generate a TSD upon integration. Three helitron-associated structural features, i.e., dinucleotide TA at the 5’ end, hair-pin loop followed by the tetranucleotide 'CTRR’ at the 3’ end, were present in two families (families 98 and 765); only the hair-pin loop could be identified in the third family (family 637). DNA transposons in total accounted for 14.6% of the repetitive DNA. Among the thirteen MITE families discovered, nine had 2-bp TSDs (TA: 8 families; CT: 1 family), two had 5 - 6-bp TSDs and one family had an 8 - 9-bp TSD. Based on having the same TIR and TSD but different internal sequences, four (families 55, 112, 222 and 290) and three MITE families (246, 298 and 2546) could be grouped into two superfamilies.

### Distribution of repeats across the chromosomes

A non-parametric runs test was used to check the randomness of repetitive sequence distribution across the *M. graminicola* chromosomes. Chromosomes were initially partitioned into bins of 100 kb each. Each bin was scored as 1 or 0, if the repetitive content of the bin was higher or lower, respectively, compared to the mean repeat content of the chromosome. A survey of these 100-kb bins showed that repetitive sequences on two core chromosomes, 8 and 10, had a nonrandom, clustered distribution. Because the number of runs was below the threshold value of 10 for all of the dispensable chromosomes and one core chromosome (number 13), we repeated the survey for smaller bins (50 kb). Using 50-kb bins, four additional chromosomes (core chromosome 6 and dispensable chromosomes 17, 18 and 19) showed a non-random distribution of repetitive sequences (Table 3; Additional file 3: Table S3). The distribution of genes also was tested in the smaller bins and showed a non-random pattern on chromosomes 8 (core chromosome) and 15 (dispensable chromosome).

**Table 3:** **Randomness of repetitive sequence distribution along each** ***Mycosphaerella graminicola*** **chromosome using a non-parametric runs test**

	Chromosome																				
Statistic	1	2	3	4	5	6	7	8	9	10	11	12	13	14	15	16	17	18	19	20	21
n	122	78	70	58	58	54	54	49	43	34	33	30	24	16	13	13	12	12	11	10	9
n1	35	26	23	17	24	22	18	18	17	12	10	13	9	6	6	5	4	4	6	6	4
n0	87	52	47	41	34	32	36	31	26	22	23	17	15	10	7	8	8	8	5	4	5
Runs	55	36	31	21	29	22^†^	19	15^†^	19	11^†^	13	13	7	10	7	6	5^†^	7^†^	5^†^	4	6
P value						2.7 e^-2^		1.9 e^-6^		1.3 e^-4^							1.5 e^-2^	1.5 e^-2^	2.7 e^-2^		

Each chromosome was divided into non-overlapping 50-kb bins. Repetitive content in each bin was compared to the chromosomal average repetitive content and each bin was scored either 1 (bin repetitive content greater than chromosomal average) or 0 (bin repetitive content lower than chromosomal average). Each consecutive occurence of 0 s and 1 s was calculated as a run. n, the total number of observations; n1, count of occurrences of ones; n0, count of occurrences of zeros; Runs, number of consecutive occurrences or runs of ones and zeros. ^†^P < 0.0.

Randomness of inter-element distances also was tested. Using the Shapiro-Wilk test, the null hypothesis that repeats are distributed randomly was rejected and the P values were highly significant for all *M. graminicola* chromosomes (results not shown). Therefore, this analysis does not seem to discriminate as well as the non-parametric means test.

### Nested blocks of elements

**Figure 4:**
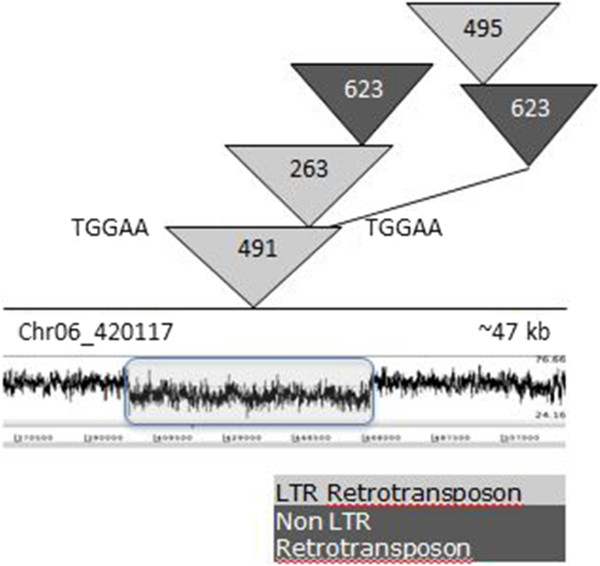
**A nested cluster of transposable elements on** ***Mycosphaerella graminicola*** **chromosome 6.** A 47-kb nested cluster with an LTR retroelement as the base is indicated. It has a 5-bp target-site duplication (TGGAA). Each triangle represents one insertion and the numbers inside the triangle refer to the repeat family. This cluster only had class I elements. These elements have been shaded as follows: light grey, LTR retrotransposons; dark grey, Non-LTR retrotransposons. The bottom panel shows the percent GC plot for the corresponding repeat region. A decrease in percent GC content in the repeat region is evident when compared to the flanking DNA sequence.

Repetitive elements inserted into other elements are described as nested. A nested cluster is initiated when a TE inserts (primary insertion event) into the base element (element in which the primary insertion occurs). There can be multiple primary insertions into the base element, as a new independent insertion event can occur adjacent to the existing insertion. A total of 78 putative nested element clusters was identified in the *M. graminicola* genome, with 69 (88%) clusters present on the core chromosomes. Their length ranged from 7.5 to 63 kb, with 33 clusters being greater than 20 kb. These clusters account for about a quarter (24%) of the total repetitive content in the genome. The number of different elements in a cluster varied from 2 to 7. An LTR retrotransposon was the base element in 49 clusters (Figure 4), out of which TSDs were verified in 35 clusters (Table 4). In eight clusters (length greater than 20 kb) with a DNA transposon or an unclassified element as the base element, TSDs were verified for the primary LTR retrotransposon insertion event. In general, the primary insertion event was dominated by LTR retrotransposons, with 59 such cases identified. One non-LTR retrotransposon family (family 623), found in 30 primary insertion events, was noticeably absent from all nested structures with a DNA transposon as the base element.

**Table 4:** **Nested clusters of repetitive elements in the** ***Mycosphaerella graminicola*** **genome with an LTR retrotransposon as base element and a verified target site duplication (TSD)**

Chr	Cluster start	Cluster end	Base family	Cluster size (bp)	Target site duplication	Internal family ^§^
1	910,151	924,049	49	13,898	GGTAG	15
1	3,757,104	3,770,311	115	13,207	CCTAC	263
2	162,839	175,222	242	12,383	CTTAG	49
2	811,149	825,327	242	14,178	CTGTG	623
2	1,675,912	1,693,177	15	17,265	CGAC	242
2	1,868,450	1,894,757	263	26,307	AAGGT/C	(2477 (495, 623))
2	3,605,891	3,620,926	15	15,035	ACAGG	623
3	575,530	603,863	940	28,333	ATTGA	(263 (623, 115))
3	787,112	832,923	495	45,811	CTAC	(2477, 623, 633 (623), 49 (115))
3	1,573,516	1,594,725	495	21,209	CCAG	(1045, 2477)
3	2,416,365	2,433,023	15	16,658	CTGC	623
4	1,534,719	1,552,726	100	18,007	ATTAG	609
4	1,567,363	1,594,101	491	26,738	ATAC	(603, 623)
5	192,764	204,730	165	11,966	TTTTG	623
6	191,008	210,165	242	19,157	ATGA	(623, 242)
6	420,117	467,482	491	47,365	TGGAA	(263 (623), 623 (495))
6	771,418	802,180	491	30,762	GGATG/GGCCG	623 (x3)
6	2,542,200	2,570,636	100	28,436	CATTG	(623 (495))
7	1,261,818	1,284,705	633	22,887	GAGCT	495
7	1,787,729	1,808,698	165	20,969	GCATC	495
7	1,821,242	1,835,308	603	14,066	GCATA/G	623
7	2,535,616	2,549,001	242	13,385	ATATC	623
9	64,037	77,995	49	13,958	GATAG	623
9	79,932	109,217	603	29,285	CATTGG	623
9	962,581	977,124	49	14,543	CCAAT	1008
9	1,052,214	1,078,572	242	26,358	TATGA	(49 (263 (623)))
10	863,891	879,094	633	15,203	GCTGC	242
10	1,306,971	1,330,127	165	23,156	AGATA	(623 (623), 623)
12	609,353	623,337	940	13,984	GCTTC	49
13	1,028,648	1,051,790	100	23,142	G/CAAAG	495
14	411,612	433,895	603	22,283	TTAAG	495
14	587,485	599,441	165	11,956	CGGTT	623
15	192,089	204,601	242	12,512	GCTTT	623
16	510,094	526,146	15	16,052	TTCT	263
18	294,431	312,546	242	18,115	GAAGA	(623, 623)

Columns: Chr, Chromosome. ^§^The brackets represent the level on nesting.

Genes also were found associated with the clusters of transposable elements. Sixteen genes (including kinases, peptidases and cytochrome c) were found inserted in the clusters and 60 genes were found in the close vicinity of these nested clusters (within 2 kb of the cluster boundary). Eleven of these genes contained signal peptides and were potentially secreted, and others included genes encoding enzymes such as hydrolases, kinases, cytochromes, and chloroperoxidases.

### Subtelomeric regions

**Figure 5:**
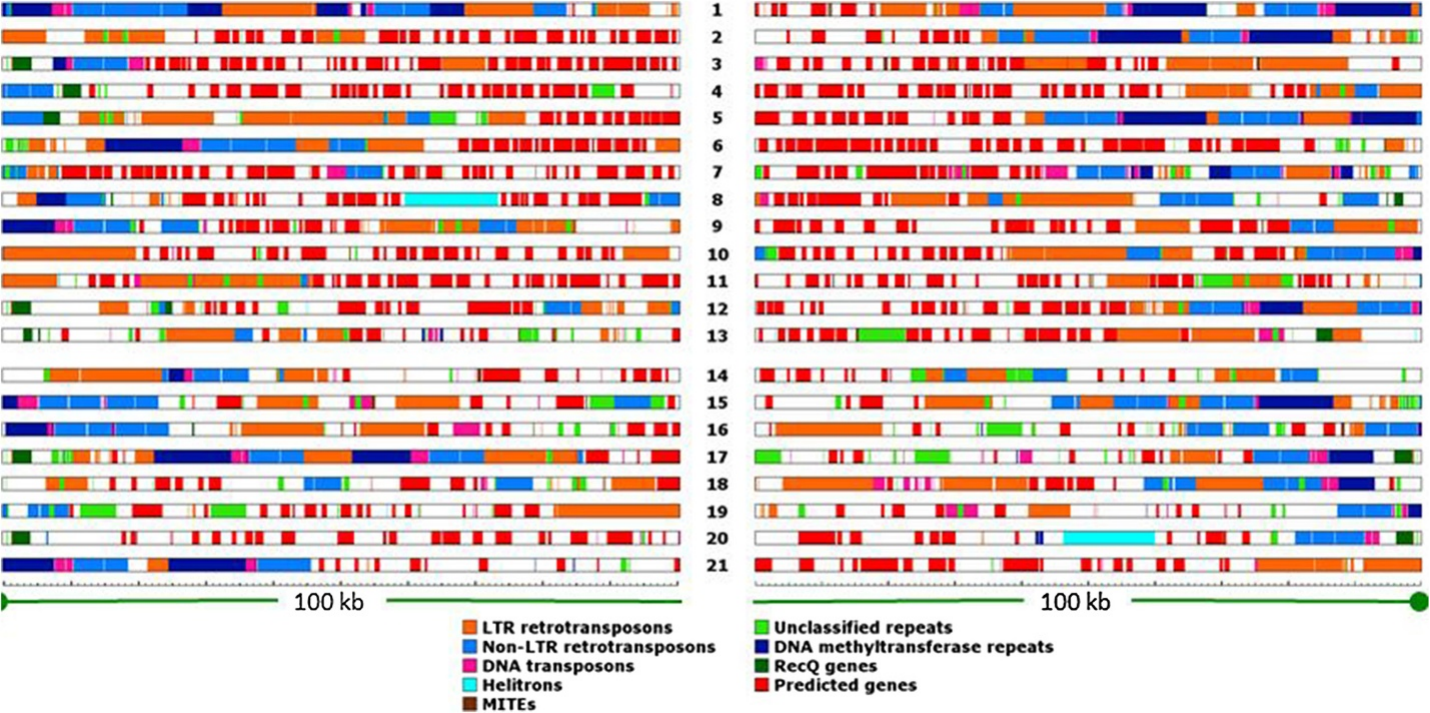
**Genes and repeats in** ***Mycosphaerella graminicola*** **subtelomeres.** A 100-kb subtelomeric sequence from both ends of each chromosome was analyzed. The numbers between the bars refer to the chromosome number. The ruler below chromosome 21 marks every 1 kb interval. All of the elements have been color coded: orange, LTR retrotransposons; royal blue, Non-LTR retrotransposons; pink, DNA transposons; brown, MITEs; cyan, Helitrons; lime green, Unclassified; dark blue, repeats containing DNA methyltransferase gene sequence; dark green, RecQ genes; red, predicted genes.

The length of a subtelomere was defined as a continuous stretch of repetitive DNA starting from the telomeric repeats, without any intervening unique DNA. Different chromosomes had varying lengths of subtelomeric repeats (Figure 5). The length of subtelomeric regions varied from 1.6% (chromosome 20) to 91.4% (chromosome 1) of the 100-kb sequence that was analyzed from the end of each chromosome. Although the repeats in subtelomeric regions did not show a biased distribution, some chromosomes had subtelomeric regions that were highly similar to sub-telomeres on other chromosomes. Long stretches of highly similar subtelomeric sequences were shared between three pairs of core and dispensable chromosomes, chromosomes 2 and 17 (58 kb), 9 and 21 (19 kb) and 4 and 18 (7 kb), and one pair of core chromosomes, 8 and 13 (37 kb).

Two repeat families were limited to subtelomeric regions only. One was a family of non-LTR retrotransposons and the other was a repeat family containing a DNA methyltransferase domain [12], atypical of repetitive elements. The non-LTR retrotransposon family (family 4) that was limited to sub-telomeric regions contained 34 full-length elements and 49 truncated elements. Depending on the number of tandem repeat copies in the non-LTR element, the full-length elements were 3.4 - 3.5 kb long. These full-length elements were present on 16 out of the 21 chromosomes and on 26 of the 41 chromosome ends. Thirteen cases were identified on different chromosomes where a truncated element was found in tandem to the full-length element. One to three copies of the telomeric hexamer repeat TTAGGG were present at the 3’ end of all the full-length and 38 truncated elements. The telomeric repeat-containing 3’ end of the non-LTR element was always pointing away from the telomere. Therefore, the orientation of the elements on one end of the chromosome was the same whereas elements on the other chromosome end were present in the opposite orientation. Telomere-associated repetitive elements with similar characteristics, i.e., containing a reverse-transcriptase gene and terminating in telomeric repeats at their 3’ ends, have been described previously as Penelope-like elements (PLEs) [19].

Besides telomere-associated repeats, one class of telomere-associated gene also was found (Figure 5). Initially, a large open reading frame (ORF) of ~ 3.2 kb was identified in the subtelomeric region of chromosome 12. Upon further annotation, the putative coding sequence was 3,480 bp long with a very low GC content of 34.7%. Similarity searches identified 13 copies (7 full length, 6 truncated) on nine chromosomes in the genome. A search of the NCBI non-redundant database revealed similarity to subtelomeric RecQ helicase from *Schizosaccharomyces japonicus* (at 5e-11). One pair of RecQ helicases was a part of the above-mentioned stretch of similar subtelomeric sequences between core chromosomes 8 and 13.

### Tandem repeats

**Figure 6:**
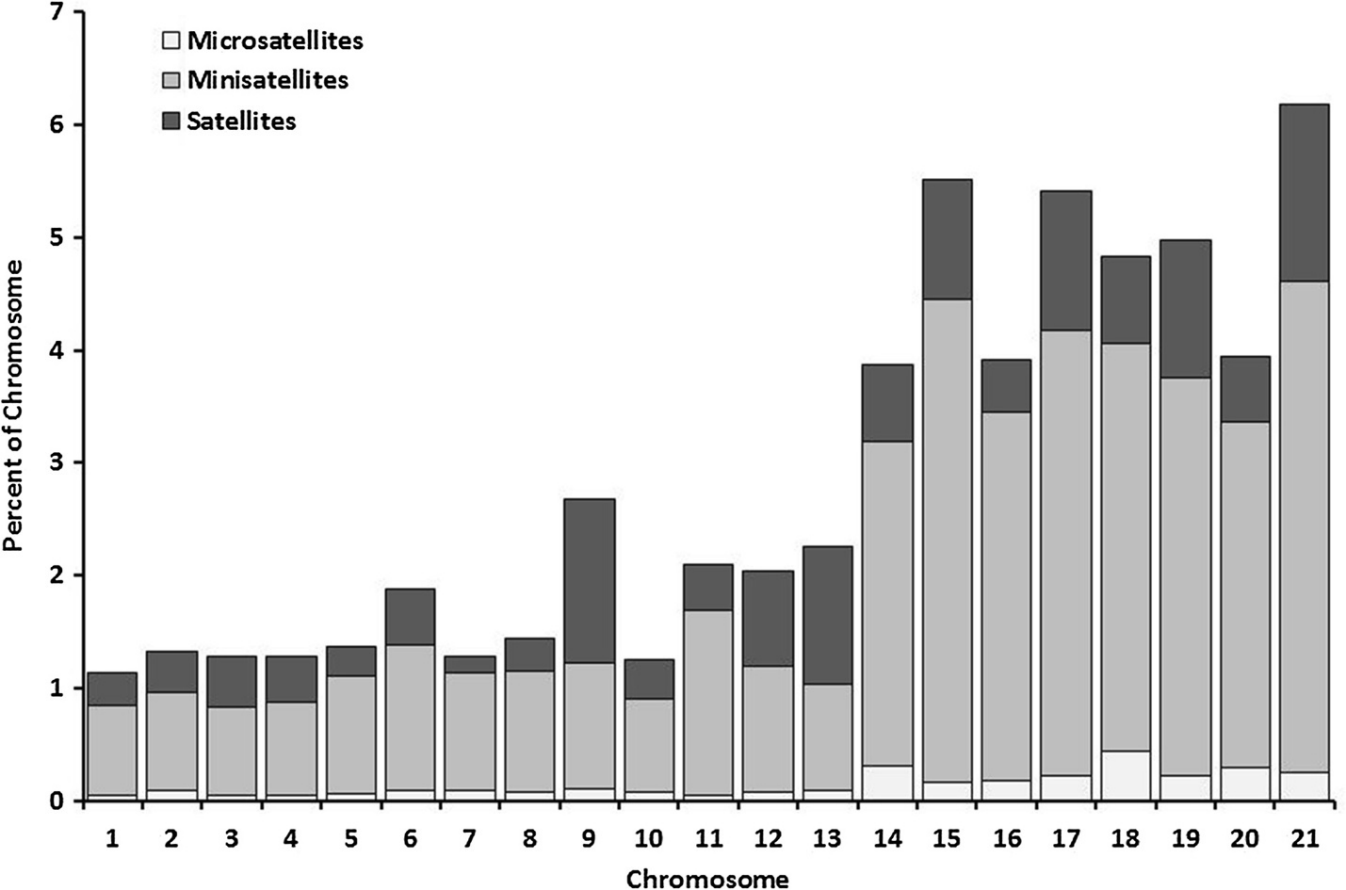
**Distribution of tandem repeats in the** ***Mycosphaerella graminicola*** **genome.** Extent of microsatellites (1–6 base pair repeat unit), minisatellites (7–100 bp) and satellites (>100 bp) across the *M. graminicola* chromosomes.

Based on their repeat unit lengths, tandem repeats are broadly classified into three categories: microsatellites (1–6 nucleotides); minisatellites (7–100 nucleotides); and satellites (>100 nucleotides). All dispensable chromosomes had a higher percentage of tandem repeats. An increase in minisatellite content contributed to this difference between core and dispensable chromosomes (Figure 6). Chromosomes 9 and 13 had an unusually higher percentage of satellite repeats as compared to other core chromosomes.

As tandem repeats often occur in genes, tandem repeat content of both unmasked and masked *M. graminicola* genome was compared. In the unmasked sequence, 225 tandem repeat families (7,509 repeats) covering 556 kb of the genome were found. The repeat unit size varied from 1 to 1950 bp. On the other hand, the number of tandem repeat families in masked sequence was reduced to 195 (5,109 repeats) and the repeat unit size varied from 1 to 479 bp. Tandem repeats in the masked genome covered a total of 374 kb with 107 kb (29%) being a part of the *M. graminicola* gene space. Thus, about one-third of the tandem repeats identified in the masked genome were components of genes, as the repetitive fraction was masked out prior to analysis.

### Extent of repeat-induced point mutations (RIP)

**Figure 7:**
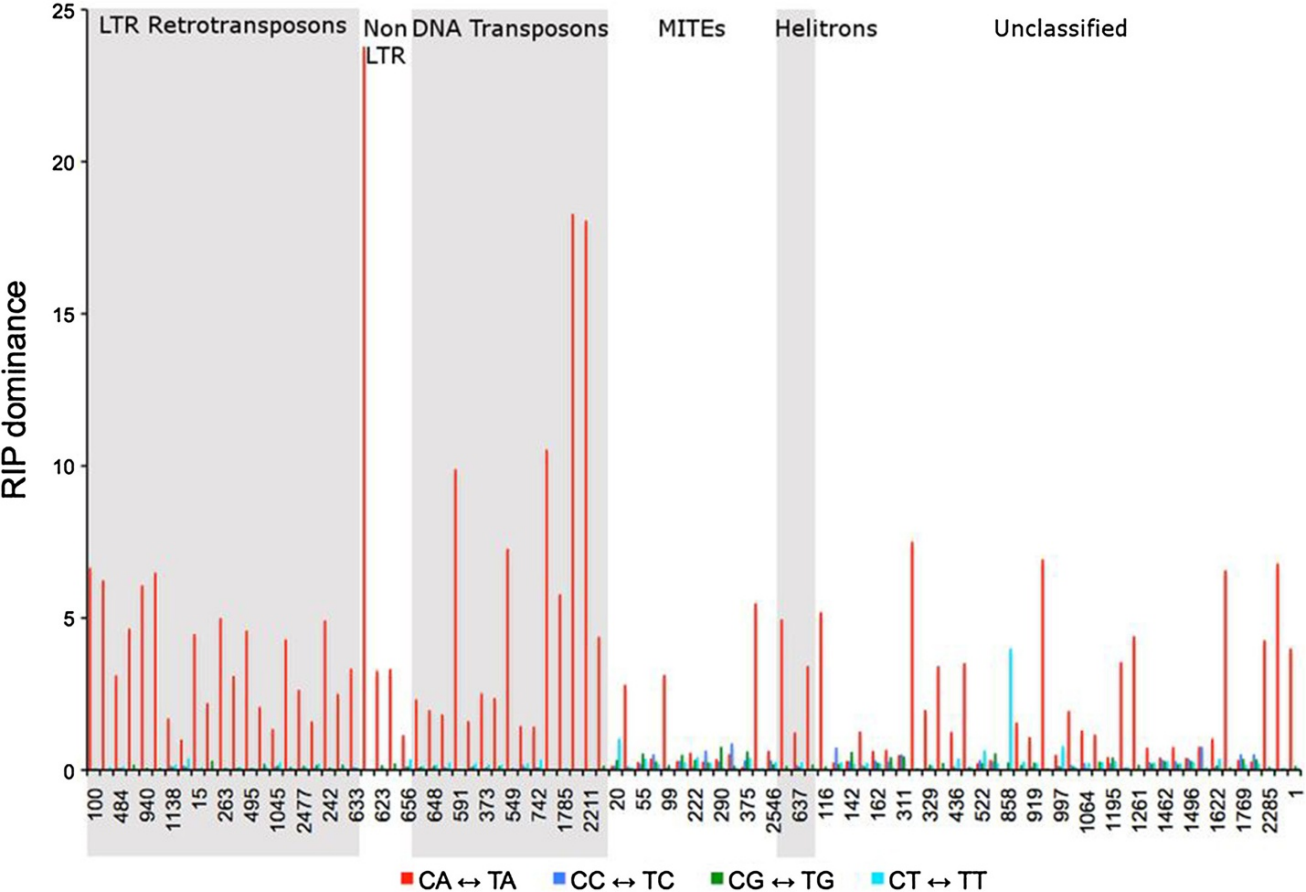
**Dinucleotide bias for the** ***Mycosphaerella graminicola*** **repeat families identified by RECON.** A specific dinucleotide bias (CpA ↔ TpA) was detected in the repeat families comprising Class I and Class II transposable elements. Ten families of MITEs and 14 of unclassified repeats showed no specific dinucleotide bias. The different dinculeotide biases that were evaluated are color coded: red, CpA ↔ TpA; blue, CpC ↔ TpC; green, CpG ↔ TpG; cyan, CpT ↔ TpT. RIP dominance, in a given alignment, is the ratio of a particular CpN↔TpN to the sum of the three other CpN↔TpN mutations. The arrows are shown bidirectionally to indicate that changes on both strands of DNA are analyzed (e.g., a C → T mutation on one strand will be seen as G → A on the complementary strand).

All repeat families showed elevated levels of transition mutations indicating RIP (Additional file 1: Table S1). A clear CpA → TpA dinucleotide bias was detected in 70 repeat families (Figure 7), while the remaining 24 families (10 MITEs and 14 unclassified repeat families) failed to show a specific dinucleotide bias. Although the analysis only looks at one strand, both C → T and G → A polymorphisms on that strand were quantified to account for RIP on the complementary strand. All of the unclassified repeats and these ten MITE families were less than 200 bp in their average match length. A pairwise comparison of repeats using the highest-GC element in each family as a reference sequence showed that the identity between elements in the same family ranged from 83 to 99%. However, two 6.7-kb sequences were identified on chromosome 7 that were 100% identical. These sequences contain the rDNA repeats, only two copies of which could be assembled in the released genome sequence.

### Active elements

Almost all elements analyzed carried numerous mutations suggesting the presence of a RIP-like mechanism in *M. graminicola*. However, elements belonging to at least one family of non-LTR retrotransposons (family 623) and one family of copia-type LTR retrotransposons (family 18) were found in the genome that carried minimal RIP-signature mutations.

Using the reverse transcriptase domain from the non-LTR retrotransposon, at least 119 similar sequences were detected in the genome. All of these sequences, except 11, carried one or more stop codons. The remaining 11 sequences also had many transition mutations, but none of them resulted in a stop codon in the reverse-transcriptase domain. These 11 elements had 90-95% sequence identity with each other and their RT domains were 97.5 - 99.9% identical. Also, a 12-bp TSD was identified for eight of the 11 sequences. This non-LTR retrotransposon is specific to fungi and belongs to the Tad-1 clade of non-LTR retrotransposons. A total of 22 repeat-derived EST sequences had 100% identity to 21 elements from this non-LTR family. Another LTR retrotransposon (family 242) was also represented in the EST dataset.

The LTR retrotransposon family with minimal evidence of RIP had only one complete copy that was present on chromosome 18. The element was 5,545 bp long with 515-bp LTRs and a 4-bp TSD (ACTT) (Additional file 2: Table S2). LTR alignment showed two mismatches, both transition mutations, which indicates a recent transposition event, as LTR sequences keep accumulating mutations with age. There was one truncated copy on chromosome 17 and two soloLTRs, one each on chromosomes 8 and 12.

## Discussion

### Repeat identification strategies

With the availability of faster and cheaper sequencing technologies, the real challenge is not sequencing a genome but its annotation. Early identification and analysis of repetitive sequences sets the ground for a better annotation of the genic and inter-genic regions. Several approaches were used for repetitive sequence analysis in the completed *M. graminicola* genome. Search strategies that exploit structural features to identify and classify TEs in sequenced genomes are known as similarity-based methods. RepeatMasker [15], a similarity-based approach, identifies repetitive elements based on their similarity to already known repetitive sequences. Besides similarity-dependent methods, two other approaches, *de novo* and k-mer based, can be used to define repetitive elements in a genome. Methods such as RECON [17] utilize whole-genome alignments to identify repetitive elements *de novo*. K-mer-based methods such as RepeatScout [18] and TALLYMER [20] calculate the frequency of oligomers of different lengths to delineate repetitive sequences in the genome. Since the k-mer and *de novo* methods do not rely on the existing repeat datasets, they are also suitable for identifying novel repeats in a genome.

With the default RepBase Update [16] repeat dataset comprising all of the fungal-specific repetitive elements only ~1% of the *M. graminicola* genome was found to be repetitive. This indicates that the RepBase Update [16] fungal repeat dataset has very low coverage of fungal-specific repetitive sequences. RepBase Update [16] has limited sequence information with 256 sequences from 56 fungal species. This low repeat estimate may also reflect that most *M. graminicola* repetitive sequences are unique, as they were not found in other species. A similarity search of thirteen sequenced fungal genomes could only identify less than 1% of the repetitive sequences present in *M. graminicola*.

RM [15] was used in conjunction with repeat libraries derived from two repeat-finding approaches, RECON [17] and RepeatScout [18]. Of these two, the RECON [17] repeat library with a cutoff for high-copy repeats of 10 members/repeat family as used previously [17] was chosen for the subsequent in-depth analysis of the repetitive content of the genome to keep the number of families analyzed in detail reasonable. However, low-copy repeat families (with 2–9 copies in the genome) also were annotated, of which only 125 families (0.05%) could be assigned a useful annotation (Additional file 2: Table S2). The output from RECON [17] is primarily used for first-pass classification of repeats in newly sequenced genomes [17]. A comparison of the repetitive fraction identified by RECON [17] alone to RECON followed by RM [15], revealed that RM was able to identify similar but divergent elements that were missed by RECON (Table 1). RepeatScout [18] output reports a consensus sequence for each family, whereas RECON [17] extracts individual elements for each family, which not only makes the annotation step easier, but also accounts for the divergent members of the repeat families. Therefore, even though RepeatScout [18] predicted a slightly higher proportion (1.9% more) of the *M. graminicola* genome as repetitive, we decided to annotate and report the RECON [17] output. The final repetitive fraction of 16.7% reported for *M. graminicola* is a conservative estimate as it excludes the low-complexity regions, simple repeats and repeat families containing fewer than 10 members/family. Adding all of those together estimated the total repetitive fraction at around 22.4% of the genome. The results from TALLYMER [20] were used for visualization purposes and correlated very well with the results from the other two methods (Figure 1). A decrease in percent GC content along the chromosome paralleled the occurrence of repetitive elements (Figure 1).

Relative insertion ages of 149 LTR retrotransposons that contained intact LTRs were estimated based on the occurrence of nucleotide substitutions between the 5’ and 3’ LTRs. However, the insertion timings may have been overestimated due to an elevated mutation rate as a consequence of RIP. Among the 149 retrotransposons with intact LTRs, only one element was found with identical sequences (insertion age of 0 My). Multiple sequence alignments of this element with other members of its family show mutated/RIPed sites in the internal region of this element but the LTRs themselves somehow escaped the effects of such mutations. This is interesting as this element would be an ideal candidate for future studies to investigate the timing and extent of RIP in subsequent cycles of sexual reproduction.

### Distribution of repetitive sequences

Analysis of a single chromosome (chromosome 7) in *Magnaporthe oryzae* revealed a non-random distribution of repeats, with repetitive sequences occurring in three clusters mostly in heterochromatic regions near the telomeres [21]. This pattern however did not hold true in the completely sequenced *M. graminicola* genome, where the actual distribution of repetitive sequences across the whole genome and all of the chromosomes could be ascertained. Repetitive sequences in the *M. graminicola* genome showed a non-random distribution across six chromosomes but were random for the remaining 15.

Distribution of repetitive sequences and of transposable elements in particular can be random or clustered depending upon the insertion preferences of the different elements. For example, LINEs and SINEs in rodents and humans show an insertion preference to distinct chromosomal domains, leading to differences in their distribution patterns in their host genomes [22, 23]. Our results show that both random and non-random distributions occur in the *M. graminicola* genome. The nested insertions primarily consisted of LTR-retrotransposons non-randomly clustered together in the gene-poor heterochromatic regions, as opposed to the smaller DNA transposons (MITEs) that were more prevalent in the gene-rich, euchromatic regions. Repeat-rich regions (AT-blocks), accounting for 36% of the genome and 5% of the gene space, are randomly distributed across the *L. maculans* supercontigs [24], whereas analyses of MITEs in the *Epichloë* genome revealed an insertion preference in the 5’ regions of the genes [25]. A dissimilar distribution pattern of repetitive elements suggests that different TEs have evolved distinct strategies to persist in the genome.

The set of eight dispensable chromosomes was statistically significantly enriched in transposable elements and tandem repeats as compared to the core chromosomes. Dispensable chromosomes, also known as B or supernumerary chromosomes, were enriched for transposable elements in other fungi, including *Fusarium oxysporum*[26] and *Nectaria haematococca*[27]. However, unlike the *M. graminicola* genome, dispensable chromosomes in these other species were enriched for genes that played a major role in plant pathogenesis.

### Types of repetitive sequences

Among all of the classes of repetitive elements, retrotransposons occupied the largest fraction in the *M. graminicola* genome. As retrotransposons follow a copy-paste mechanism for replication, these elements have usually been the major contributors to the repetitive fraction across a large number of genomes analyzed so far. The role of Class I retrotransposons in genome size inflation has been studied extensively in various organisms. In plants, a single family of LTR retrotransposon, BARE-1, is positively correlated to barley genome size increase [28], whereas genome size doubling of the wild rice *Oryza australiensis* was attributed to three LTR retrotransposon families that accounted for 60% of the genome [29]. Retrotransposons are also implicated in genome size expansion in fungi and oomycetes. The two Dothideomycetes relatives of *M. graminicola* with expanded genomes, *Cladosporium fulvum*[30] and *M. fijiensis*[31], had higher proportions of LTR retrotransposons in their genomes. The genome of the powdery mildew pathogen *Blumeria graminis* is four times larger than the average ascomycete genome; this difference can be attributed to non-LTR retrotransposons [32]. Similarly, in the oomycete *P. infestans*, two LTR retrotransposon families account for 29% of the 240-Mb genome [9].

The *M. gramincola* retrotransposon families showed various stages of decay. There were six LTR retrotransposon families in which LTRs or TSDs could not be identified. The remaining LTR retrotransposon families also had truncated elements. Fragmented sequences that lack any retrotransposon structural features but show similarity to partial retrotransposon sequences have been termed 'remnants’ [33]. Such remnants with small or large deletions have been observed in other organisms. Various mechanisms, such as illegitimate recombination in *Arabidopsis*[34], *Saccharomyces cerevisiae*[35] and *Drosophila*[36] and unequal homologous recombination in plants [33], are responsible for deletions in repetitive elements and tend to counteract the repetitive element expansion in the genome. The extent of remnants in a family is correlated to the age of the family [33], with older families having a larger number of truncated elements.

Three putative helitron families carrying helicase domains were identified but only two families had the hallmark structural features associated with helitrons. Occurrence of truncated elements in the third family made the identification of structural features impossible. Helitrons have been detected in other ascomycete fungi, such as *Aspergillus nidulans*[37] and *Dothistroma pini*[30]. An expansion of helitrons was observed in the *P. infestans* genome, which had 10-fold higher copies than the related *P. ramorum* and *P. sojae* genomes [9]. As compared to MITEs, TIRs and TSDs could not be identified for any of the DNA transposon families, which suggests that these transposon families may be old. Crypton, a class of DNA transposons found only in fungi [38] was also present in *M. graminicola*. These crypton sequences were initially identified by RECON [17] but were not included in the parsed set as they were below the family cut-off threshold of at least 10 elements.

### Telomeres, RecQ helicase and putative Penelope-like elements

The RecQ helicase gene family, with extremely low GC content and tightly linked to the *M. graminicola* telomeres, has previously been identified in other fungal genomes including *M. grisea*[39], *S. cerevisiae*[40] and *Ustilago maydis*[41]. RecQ helicases are involved in a variety of functions including post-transcriptional gene silencing [42], maintaining genome stability [43] and DNA repair and recombination [44]. In *S. cerevisiae*, RecQ helicase is a part of the telomere-localized Y’ element [45], which has been shown to play a structural role in protecting telomeres from accidental shortening or damage from recombination-mediated mechanisms. In *S. cerevisiae* strains lacking a gene for telomere replication, amplification and acquisition of Y’ elements in a large number of telomeres decreases the frequency of cell death [46]. Besides a structural role, yeast RecQ helicase expression is induced during meiosis, suggesting a role in meiosis or sexual development [40].

Penelope-like elements (PLE), characterized by a bacterial GIY-YIG endonuclease domain, have a widespread distribution across the tree of life including other fungi, rotifers, plants and heterokonts [47]. One family of telomere-localized PLEs was identified in the *M. graminicola* genome. These putative PLEs are a subtype that localize to the subtelomeric regions in a characteristic orientation with telomeric repeats at their 3’ ends [19]. Elements of this subtype lack the endonuclease domain but instead have species-specific telomeric repeats at their termini that can pair with the single-stranded telomere and utilize the 3’ hydroxyl group for priming sequence synthesis [19]. Thus, these telomeric PLEs may play a role in protecting telomeres in addition to the regular telomerase enzyme, which in *M. graminicola* is located on chromosome 7. Telomeres in *Drosophila*[48], silkworm [49] and *Giardia lamblia*[50] are also maintained by non-LTR elements, but they carry an endonuclease domain. The telomeres in these three species consist of long arrays of non-LTR elements, unlike the endonuclease-lacking PLEs which are low-copy repeats.

### RIP in repetitive sequences

Due to their inherent ability to amplify, defense mechanisms exist in the genome to minimize the numbers of transposons. One such mechanism that is specific to fungi is Repeat-Induced Point mutation (RIP). RIP targets multiple-copy sequences during meiosis and introduces cytosine (C) to thymine (T) mutations [51]. Sexual reproduction in the field is a common phenomenon in *M. graminicola* and ascospores have been shown to play a major role in long-distance disease spread and initiation [52]. Therefore, it seems highly likely that repetitive sequences in *M. graminicola* can be targeted by RIP during meiosis. Although RIP was not verified experimentally, a detailed analysis for all the repeat families revealed a higher number of transition mutations, supporting the existence of a RIP-like mechanism in *M. graminicola*, as noted previously [12, 13]. However, our results were based on a much larger and global dataset as compared to the previous reports.

Certain dinucleotides are preferentially targeted by RIP, known as RIP bias, which varies with the organism [53]. Repetitive sequences in *M. graminicola* exhibited a CpA dinucleotide bias, the same as seen in *Neurospora crassa*[53]. However, ten families of MITEs and 14 families of unclassified elements did not show any clear dinucleotide bias. Repeat elements in these families were shorter than the minimum length threshold detected by the RIP machinery. RIP typically acts on sequences that are longer than 400 bp in length [51] and are at least 80% identical [54]. Many families contained truncated elements and fewer full-length elements that might have prevented the detection of RIP bias *in silico*. Two completely identical *M. graminicola* rDNA repeat copies suggest that they were not detected by RIP. The presence of identical rDNA sequences is expected as these repeat clusters can evade RIP, although the exact mechanism is unknown [55].

The presence of transition mutations in the *M. graminicola* repetitive sequences generated many stop codons in the putative repeat-encoded protein domains. In *N. crassa*, RIP increased the occurrence of termination codons TAG and TAA [56]. However, a few elements in at least two families (families 623 and 18) were identified with no stop codons, although the presence of transition mutations in other parts of the elements indicated that the RIP machinery targeted these sequences. Lack of stop codons in the reverse-transcriptase domain suggested that these elements might still be active in the genome. Evidence for activity is also available at the transcript level, with at least two repetitive families (families 623 and 242) represented in the *M. graminicola* EST dataset. Therefore, these elements might be functional and active in the genome until they can be identified and targeted by the RIP mechanism during sexual reproduction.

### Nested clusters of transposable elements

In a number of eukaryotic genomes, TEs are nested or inserted into other TEs [57, 58]. Although many classes of TEs target specific regions in the genome, no obvious insertion preference for elements was identified in *M. graminicola*. As LTR retrotransposons were the most abundant in the *M. graminicola* genome, the majority of nested clusters identified had an LTR retrotransposon as the base element. One of the mechanisms by which LTR retrotransposons can target specific regions in the genome has been analyzed in *Saccharomyces cerevisiae*[59]. In *S. cerevisiae*, the Ty5 LTR retrotransposon targets telomeric heterochromatin as a result of direct interaction between retrotransposon-encoded integrase protein and the *silent information regulator 4* protein (Sir4), associated with heterochromatin [59]. Nested clusters also have been reported in other fungi such as *L. maculans*[24] and *Epichloë* species [60], but were absent from other expanded fungal genomes, such as *B. graminis*[32]. Nested clusters also have been mentioned in *F. oxysporum*[61], *N. crassa*[62] and *M. grisea*[63–65], but they are mainly in the putative centromeric regions. The centromeres of *M. graminicola* have not been identified so whether they contain repeats is not known.

Due to their larger genome size and higher repetitive content, nesting of TEs has been studied extensively in plants. In maize, where 85% of the genome is repetitive [66] and ten retrotransposon families make up more than 25% of the genome, nested clusters may be a way to reduce the deleterious effects of TEs [57]. Genes were found in and near these nested TE clusters. Proliferation of a specific pathogenicity factor gene family unique to the powdery mildew pathogen *B. graminis* has been attributed to a family of LINE retrotransposons [67]. Such gene-repeat associations may hold an evolutionary and functional significance to the *M. graminicola* genome as it has been previously shown that in fungal and oomycete genomes, such repeat clusters act as 'breeding grounds’, where new pathogenicity genes can be generated [9, 24, 30].

## Conclusions

Repetitive sequence analysis revealed that at least 16.7% of the *M. graminicola* genome was comprised of repeats. Dispensable chromosomes had a significantly higher repeat content as compared to core chromosomes. Class I elements occupied the largest fraction of the repetitive sequences in the *M. graminicola* genome. One family of telomere-localized Penelope-like elements was identified in the *M. graminicola* genome. The distribution of repeats was non-random on six chromosomes. Repeats in *M. graminicola* often were arranged in nested clusters of 2–7 elements. Even though all the transposable elements were riddled with transition mutations, there were putative transcriptionally-active elements in the *M. graminicola* genome as inferred from the absence of stop codons in EST sequences corresponding to at least three repeat families in the EST dataset.

## Methods

### Identification of repeats

Three methods were used to identify the repetitive sequences in *M. graminicola*. Repeats were identified *de novo* using RECON [17] and RepeatScout [18], a k-mer based method. The output from these two programs was used to generate custom repeat libraries, which were subsequently used to mask the *M. graminicola* genome using RepeatMasker [15]. Manually curated repeats specific to fungi were obtained from RepBase Update [16]. Repeats were also identified using another k-mer approach, TALLYMER [20], and frequencies of repetitive sequences were plotted along the chromosomes using Gnuplot [68].

### Annotation of repeats

The repeat families identified by RECON [17] were annotated. The default output from RECON [17] was parsed to include families with 10 or more elements. The longest element from each family was compared against the NCBI non-redundant protein database using BLAST [69] to identify protein domains. These protein domains were used to classify repetitive sequences into different classes. Structural features such as Long Terminal Repeats (LTRs) and Terminal Inverted Repeats (TIRs) were verified in LTR retrotransposon and DNA transposon families using POLYDOT and EINVERTED, respectively, both from the EMBOSS [70] package. TIRs were also identified for families with no known protein sequences to identify Miniature Inverted Repeat Transposable Elements (MITEs). Sequences with no known proteins or other structural features were grouped into the unclassified category.

### Insertion age estimation for LTR retrotransposons

Pair-wise alignment of intact LTRs from each LTR retrotransposon was used for estimating the age of insertion events. Numbers of substitutions were calculated for each pair and translated into divergence time using a substitution rate of 1.05 × 10^-9^ nucleotides per site per year, as previously determined for fungi [71, 72].

### Distribution of repeats

A non-parametric runs test for randomness was used to determine whether the repetitive sequences on the chromosomes occur in a pattern or are random. Each chromosome was divided into non-overlapping 100-kb bins and the extent of repetitive bases in each bin along with the average repetitive content/bin for the chromosome were calculated. The Lawstat package [73] in R [74] was used to do the analysis. The above steps were also repeated for non-overlapping, smaller 50-kb bins. A similar analysis for gene distribution was done using 50-kb bins.

### Estimation of RIP

Elements in each family were aligned using clustalX [75] and the alignments were manually curated using Jalview [76]. These alignments were used for estimating RIP dinucleotide bias using RIPcal [77]. For calculating percent identity, pairwise comparisons between elements of 15 repeat families were done. Elements with the highest GC content in each family were used as reference sequence. Sequence pairs with at least 99% sequence coverage were evaluated.

### Nested elements

Relative chromosomal locations of the elements and their family annotations were used to identify clusters of nested elements. The base element was defined as the element into which all others were inserted. The alignment file for the base element family was used to determine if different parts of the base element made up a complete element. Target site duplication (TSD) of the base element in a given cluster was identified manually by examining the sequence in ARTEMIS [78].

### Subtelomere organization

The distribution of different repetitive elements and proteins was analyzed over a 100-kb sequence from each end of the 21 chromosomes and viewed with OmniMapFree (http://www.omnimapfree.org). Repetitive family annotation was used to check for families exclusive to subtelomeric regions. For annotation of telomere-associated repeats and gene families, BLAST searches against the *M. graminicola* and NCBI 'nr’ databases were done. The non-LTR retrotransposon reverse transcriptase domain was used to classify subtelomeric non-LTR retrotransposons using a web-based tool, RTclass1 [79].

### Tandem repeats

Tandem Repeat Finder [80] was used to do whole-genome analyses to identify tandem repeats. Both the unmasked and masked (RM 'nolow’ option) *M. graminicola* sequence was used for this analysis. The results were parsed using Tandem Repeat Analysis Program [81].

### Availability of supporting data

The data sets supporting the results of this article are available in the LabArchives, LLC, repository, DOI 10.6070/H4222RRG [unique persistent identifier and hyperlink to dataset(s) in http:// format will be submitted and provided if accepted].

### Endnote

Names are necessary to report factually on available data. However, the USDA neither guarantees nor warrants the standard of the product, and the use of the name implies no approval of the product to the exclusion of others that also may be suitable.

## Electronic supplementary material

Additional file 1: Table S1: Repetitive families identified in *Mycosphaerella graminicola* by RECON. A summary of the general features of repetitive families identified in the finished genome sequence of *M. graminicola* by RECON. These features include family number, family name, element copy number, base pair coverage, annotation, families that were merged together, LTR/IR length, TSD length, presence/absence of RIP and RIP index value. (XLSX 60 KB)

Additional file 2: Table S2: Repetitive families with fewer than 10 repeat elements per family identified in the *Mycosphaerella graminicola* genome. Repeat classification for 125 repetitive families, which had fewer than 10 elements per family in the *M. graminicola* genome. (XLSX 64 KB)

Additional file 3: Table S3: Runs test for randomness of repetitive sequences across the *Mycosphaerella graminicola* chromosomes. This table lists the consecutive runs of 0 s (repetitive content in a non-overlapping 50-kb bin less than the chromosomal average) and 1 s (repetitive content of the 50-kb bin is above the chromosomal average). (XLSX 65 KB)
